# Communicative challenges among physicians, patients, and family caregivers in cancer care: An exploratory qualitative study in Ethiopia

**DOI:** 10.1371/journal.pone.0230309

**Published:** 2020-03-13

**Authors:** Bethlehem Girma Kebede, Aynalem Abraha, Rune Andersson, Christian Munthe, Mats Linderholm, Barbro Linderholm, Nataliya Berbyuk Lindström

**Affiliations:** 1 Department of Applied Information Technology, University of Gothenburg, Gothenburg, Sweden; 2 Department of Surgery, School of Medicine, Health Science College, Addis Ababa University, Addis Ababa, Sweden; 3 Department of Infectious Diseases, Institute of Biomedicine, the Sahlgrenska Academy, University of Gothenburg, Gothenburg, Sweden; 4 Department of Philosophy, Linguistics and Theory of Science, University of Gothenburg, Gothenburg, Sweden; 5 Ersta Hospital, Stockholm, Sweden; 6 Department of Oncology, Institute of Clinical Sciences, the Sahlgrenska Academy, University of Gothenburg and the Sahlgrenska University Hospital, Gothenburg, Sweden; University of Auckland, NEW ZEALAND

## Abstract

**Background:**

Cancer is a growing concern in Ethiopia. Though communication is essential for the treatment process, few studies have looked at communication in Ethiopian cancer care. Due to the large number of patients and scarcity of resources, it is vital to understand how to manage consultations in order to effectively help as many patients as possible in this challenging work environment. Thus, research is needed to analyze and understand the communicative challenges experienced by physicians, patients, and family caregivers, in order to successfully handle patient care in practice.

**Objective:**

We explore communication in Ethiopian cancer care and present the main challenges faced by physicians, patients, and family caregivers.

**Methods:**

This explorative qualitative study was conducted at the Oncology Department of the Tikur Anbessa (Black Lion) Specialized Teaching Hospital (TASH) in Addis Ababa, Ethiopia. A triangulation of data collection methods was used: 91 audio-recorded, semi-structured interviews and 21 video-recordings of authentic interactions during hospital rounds. The aim was to obtain as complete a picture as possible of communication from the perspectives of physicians, patients, and family caregivers. The interviews were analyzed using thematic content analysis and the identified themes were supported by excerpts from the transcribed recordings.

**Results:**

Eight themes emerged from the data. Workload and time pressure, in combination with restricted space for privacy, limited the possibilities for physicians to deliver detailed information and provide emotional support. Furthermore, patient literacy levels, in combination with no or little cancer awareness, financial problems, reliance on traditional and religious treatments, the stigma of cancer, and a fatalistic attitude, resulted in delays in patients seeking care and participating in positive health behaviors, and, subsequently, often resulted in an unwillingness to openly discuss problems with physicians and adhere to treatment. The study also illustrates the paramount role of family in physician-patient communication in Ethiopia. Though family caregivers provide a valuable interpreting support when patients have limited language skills, they can also prevent patients from sharing information with physicians. Another important finding is that family caregivers were often responsible for making decisions about treatment and avoided telling patients about a poor prognosis, believing that conveying bad news may upset them. All of these themes have important implications for the role of ethically acceptable communication in patient-centered care.

**Conclusions:**

This study has identified a number of serious challenges for successful and ethically acceptable health communication in Ethiopian cancer care. The study contributes to our understanding of the complexity around the role of family, combined with patients’ dependency on family members for communication, support, and access to care, which creates particular ethical dilemmas for the medical staff. The questions raised by this study concern how to organize consultations to achieve patient-centered health communication, while maintaining a constructive alliance with the family and not jeopardizing the patient’s continued access to care. The integration of communication training for medical students in Ethiopia, with a focus on ethical guidelines for family-centered patient consultation suitable for these circumstances, would be an essential step.

## 1 Introduction

Though cancer and other non-communicable diseases are a growing concern in Ethiopia and Sub-Saharan Africa [1–3], Ethiopia’s healthcare resources are currently primarily directed at the prevention and treatment of communicable diseases, such as malaria and diarrhea [4]. Doctors and nurses are in short supply and oncology services are scarce [5, 6]. The Oncology Department of the Tikur Anbessa (Black Lion) Specialized Teaching Hospital (TASH) in Addis Ababa is the country’s sole specialist unit for cancer care. Currently, it treats about 10 000 patients per year, while the estimated annual incidence of cancer is over 60,000 cases and the annual mortality is over 44,000 cases [4, 7, 8].

The hospital’s Chemotherapy and Radiotherapy Center is the only facility that provides radiotherapy services to cancer patients in Ethiopia [9]. The working conditions are challenging, with few senior oncologists, mainly junior physicians, a handful of nurses, a lack of resources (at the hospital as well as among patients), and an enormous influx of patients from all over the country. This primarily affects the prerequisites for effective and ethical patient consultations. Patients wait for weeks to see a doctor and many depart without having received any therapeutic or preventive assistance. They are usually accompanied by family members or other caregivers, who are present when initial information is given, the results from the diagnosis are addressed, and treatment options are discussed. Handling these meetings effectively is essential in order to help as many patients as successfully as possible within the limitations mentioned, and to achieve ethically acceptable health communication and patient-centered care [10, 11]. Thus, research is needed to understand the communicative challenges involved in order to handle meetings between doctors, patients and caregivers more effectively in practice and to provide high-quality care [12].

Few studies on communication in Ethiopian health care are available in general, and in cancer care in particular [13, 14]. Cancer awareness and knowledge in Ethiopia is low, especially relating to cervical cancer [15], but also regarding breast cancer [16]. In addition, the lack of a national screening system and low access to relevant healthcare service reportedly contribute to inefficient testing and late diagnosis and treatment, which results in many women seeking help too late [14, 17–19]. There are also barriers that prevent many Ethiopians from seeking medical help such as a sense of hopelessness and anxiety [9], a fatalistic attitude [20], stigmatization and social isolation in relation to particular diagnoses (for instance, the etiology of cervical cancer is thought to be due to breaching social taboos or engaging in unacceptable behaviors [18]), ignoring early signs and symptoms [21], a lack of resources to cover treatment costs, and a tendency to turn to traditional healers in the first instance [14, 22].

Patients’ families play an important role in the treatment process. Besides covering travel and treatment costs [6, 23], a patient’s family plays an important role in decision making and especially in breaking bad news [24]. In Ethiopia, in the case of terminal illness, the disclosure of bad news to patients is discouraged by family members, who often believe that it may lead to unnecessary distress, loss of hope, and worsen their ill relative’s condition [25].

The aim of this study is to explore communication in Ethiopian cancer care from the perspectives of physicians, patients, and family caregivers.

## 2 Material and methods

### 2.1 Study design

The study was conducted at the Chemotherapy and Radiotherapy Center of TASH in Addis Ababa, Ethiopia from 2015–2019. The center was established in 1997 in collaboration with the International Atomic Energy Agency (IAEA). The center’s resources include 5 oncology doctors, 16 oncology nurses, 3 medical physicists and 4 radiotherapy technologists, and there are 2 radiotherapy treatment machines (a Cobalt 60 and a Linear Accelerator). In the admission ward in TASH, 18 beds are provided and 17 beds are available in a satellite clinic (35 beds in total). The center began training clinical oncologists in 2013, and currently has 36 enrolled residents. Training of oncology nurses commenced in 2015. The activities of the center include an inpatient service for chemotherapy and outpatient clinics for new patient evaluation and follow-up of patients under treatment. The center is linked to the Oncology Department at Addis Ababa University and is the only facility available to provide specialized services for cancer patients in Ethiopia [45].

To study the communication situation in this context, we adopted an ethnographic explorative qualitative study design, using a triangulation of data collection that combined semi-structured interviews with direct observations and video-recordings of authentic interactions between physicians, patients, and family caregivers during hospital rounds. Triangulation helped to provide as complete a picture as possible of the challenges associated with communication in cancer care and increased the possibility of understanding this complex phenomenon [26] from the perspectives of central stakeholders.

### 2.2 Sampling and participants

The participants in the study (physicians, patients, and family caregivers) were purposively sampled [27] in order to get a heterogeneous sample in terms of gender, cancer types (the patients), and work experience (the physicians). This was done in order to minimize the risk that important perspectives linked to such background factors were left unexplored by the study.

Sixteen physicians (3 senior and 13 junior, n = 16, 11 male and 5 female, age range 29–58 years), who had regular contact with patients, were approached during staff meetings in their workplace at TASH. All the physicians who were contacted agreed to participate in both interviews and video-recordings. The participants among patients and family caregivers were chosen in collaboration with the nurses who booked their consultations. Patients whose clinical symptoms made it questionable or impossible to participate in the interviews, as well as patients or relatives suffering stress, anxiety, and cognitive impairments, were excluded from the study. Since people experiencing the most stress were excluded from the study, some of the most serious needs may have been overlooked.

In total, 54 patients (20 male and 34 female, age range 22–53 years) diagnosed with cancer and undergoing treatment at TASH and their family caregivers (n = 22, 11 male and 11 female) were approached in the waiting area of the hospital by Kebede. The participating patients had the following types of cancer: breast, cervical, intestinal, rectal, oral cavity, esophageal, lacrimal gland, nasopharyngeal, and Hodgkin lymphoma, and came from both rural and urban areas.

First, the interviewer explained the purpose of the study and invited patients to participate in the interviews and asked for permission to audio-record them. All patients and their family caregivers gave their oral and written consent and signed the interview consent forms both before and after the interviews.

Next, at the end of each interview, the patients and family caregivers were asked for permission to video-record their communication with their physicians during hospital rounds. All 16 physicians, as well as 21 patients, and 20 family caregivers agreed to participate in video-recordings and signed the consent forms for them. The main reasons for refusing to participate were uneasiness in front of a camera and lack of time. Once agreement was obtained, a small video camera was placed outside the participants’ field of vision to record the interactions between physicians, patients, and their family caregivers. Kebede and Berbyuk Lindström were not present during the recordings of patient consultations to minimize the effect of filming on the participants’ behavior [28]. No one other than the participants who signed consent forms were recorded. The physicians and nurses (if present) took responsibility for covering the camera lens when necessary (for example, in the case of physical examination), or turning the camera off upon request by patients or family caregivers.

The consent forms were written in Amharic and interpreted in Oromo, if necessary, by Kebede and medical staff, and were read aloud to patients and family caregivers with low literacy skills.

### 2.3 Data collection

The data collected included audio-recorded, in-depth, semi-structured interviews with 54 patients, 21 family caregivers and 16 physicians. The interviews with patients and family caregivers were conducted in Amharic and the interviews with physicians were conducted in English, and all interviews were conducted on TASH premises. Kebede conducted and audio-recorded the interviews using an interview guide developed together with Berbyuk Lindström, based on health communication theory and standard concerns in cancer care [29, 30], as well as consultation with clinical staff at the study site to ensure feasibility and relevance (see Interview Guide). The questions were related to the participants’ experience of communication in relation to cancer care, the challenges experienced, and ways of handling them. Finally, all the participants were encouraged to comment on the interview questions and add any additional information that they wished to contribute. The average interview length was fifteen, nine, and eleven minutes for the physicians, patients, and family caregivers respectively. Total interview time was approximately four hours for the physicians, eight hours for the patients, and three and a half hours for the family caregivers. Due to the workload of the physicians and the volume of patients, it was not possible to conduct longer interviews without disturbing routines.

Most patients and family caregivers were very shy and gave short answers. A possible reason for this could be that some patients and caregivers were unwilling to express criticism fearing their relationship with the treating physician might be negatively affected.

In total, 21 hospital round interactions were video-recorded. The mean recording time was seven minutes and total video-recording time was two hours and twenty-one minutes.

### 2.4 Ethics approval

The context of the study (a patient ward for seriously ill people in an environment of very limited resources), and the nature of the data collected (detailed sensitive personal information in both text, sound and moving image) necessitated careful ethical consideration, and legally mandated formal ethics review board approval. An ethical basis for all health-related research is that the potential scientific and “social” value of the research balances the ethical challenges [31]. The pressed situation in Ethiopian cancer care (described above) and the lack of research and systematic analysis of the communicative, ethical, and quality of care implications motivates further studies. In other words, the many challenges present in Ethiopian cancer care creates a situation of “clinical equipoise”, which is a standard criterion for justifying clinical research [32]. To ground a substantial first analysis with a “social value” potential, it is important that not only relevant testimonies are collected (interviews), but also that health communicative situations are directly documented (video-recordings). One ethical challenge with this method is not to unnecessarily burden patients and relatives with the data collection. This challenge was handled by our process of informed consent (described earlier), by researchers not being present during video-recordings of consultations, and by excluding patients with very serious symptoms, as well as patients and relatives suffering cognitive impairment or situational factors that may undermine decision capacity (such as stress and anxiety). Another challenge is to ensure sufficient protection of data that contains many layers of sensitive personal information about vulnerable people, and is by its very nature impossible to de-identify. This challenge was handled by meeting standard requirements from ethical review boards regarding protected storage of sensitive raw data in a secure location. Access to this data is limited to authorized persons, with ethical review boards serving as authorizing bodies. Informed consent from participants has been collected on this condition (See the Data Availability statement for information about how researchers may obtain authorization to access the data.). All of these considerations and measures were included in applications for approval to ethical review boards in Sweden and Ethiopia. Ethics approval was obtained from the Ethical Review Board of TASH (04/14/2015) and the Ethical Review Board of Western Sweden (DNR 520–18).

### 2.5 Data analysis

The analysis employed a mostly inductive analytic approach, with interdisciplinary theoretical background material from communication theory, bioethics, global health and clinical oncology care to ground interpretative hypotheses, and analysis of further implications.

The audio-recorded interviews were transcribed verbatim and translated into English. Thematic content analysis [33] was used for data analysis. Using this qualitative technique to analyze our data involved an iterative process of listening to the interviews and reading the transcriptions, assigning codes, and finally determining patterns in the material. The codes were identified independently by Kebede and Berbyuk Lindström, who read the transcripts separately several times and then discussed the codes assigned. The coding categories that had a high degree of agreement between the coders were then discussed and sorted into themes. Trustworthiness of these hypotheses was evaluated through interdisciplinary deliberative discussions in the researcher group, as well as seminar presentations of preliminary findings. Finally, in collaboration with the other authors, the themes were reviewed. Representative quotes for each theme are presented in the results section below.

The video-recordings were translated from Amharic into English, and transcribed using a simplified version of the Gothenburg Transcription Standard [34] by Kebede and checked by Berbyuk Lindström. Kebede and Berbyuk Lindström independently read the transcripts and identified the sequences in interactions to illustrate the themes mentioned in the interviews. Both verbal and non-verbal communication were considered in the analysis. The excerpts from the transcripts illustrate the themes identified in the interviews.

The combination of interviews with video-recordings of authentic interactions allows for gaining an understanding of the participants’ perspectives on communication and obtaining insights into how they communicate in practice. By illustrating themes with illuminative quotations and excerpts from interactions, confirmability was ensured, providing a clearer understanding of the communication challenges in cancer care.

## 3 Results

The findings reveal eight main themes relating to communicative challenges in oncology care. These include:

Theme 1. Workload and time pressure

Theme 2. Lack of privacy

Theme 3. Managing language problems

Theme 4. Delivering bad news

Theme 5. Decision making

Theme 6. Illiteracy and cancer awareness

Theme 7. Traditional and religious treatments

Theme 8. Stigmatization and fatalism

The physicians reported that many communicative challenges stem from the unfavorable combination of an extreme influx of patients and a shortage of physicians, which results in an overwhelming workload and difficulties in allocating sufficient time to their patients and family caregivers (Theme 1. Workload and time pressure). The physicians mentioned that they are aware of not providing all information to patients and family caregivers and that some information is underprioritized:

*Doctors tell patients everything, for example, the side effects*. *This approach is not commonly applied in Ethiopia*. *But in practice it should happen. (Dr. male 9)*

Patients and family caregivers were also frustrated at not being given sufficient consultation time. Though many described their communication with physicians positively, some were concerned about not getting enough information. A female family caregiver commented:

*Most of the doctors are very nice and humble when they speak to you*. *But when you ask them information about the diseases, they don’t usually give you adequate information*. *I knew nothing about cancer before, and this is the right place to get information, but they don’t give you enough*. *Maybe they don’t have enough time. (Careg. female 31)*

Lack of time becomes especially challenging in relation to breaking bad news and managing emotional reactions (Theme 4. Delivering bad news), as many physicians simply have no time to provide emotional support to their patients:

*The doctor told me that “you know your patient has reached the fourth stage*. *She was supposed to take chemotherapy three weeks after the operation.” First, I didn’t know anything about the cancer stages*. *Second nobody has informed my patient or me about the chemotherapy after the operation*. *All the information we obtained was to wait six months in the queue*. *After the bad news I felt terrible, couldn’t control myself, all the emotions came at once and we didn’t get any help from the doctor… that was so devastating. (Careg. female 3)*

Another factor influencing communication was lack of privacy (Theme 2. Lack of privacy). The patients in the outpatient department were often examined in a small office-type room with one stretcher, and other people, in addition to the physician, nurse, and family caregivers, might be present. As other people could hear sensitive information, it creates stress and an unwillingness in patients and family caregivers to disclose their concerns freely. Other staff and patients could also interrupt interactions at any time:

*Either nurses or other patients always interrupt them (doctors) while they are talking to you*. *(Careg. male 10)*

Interrupting interactions can be especially problematic when patients and physicians experience language barriers in communication (Theme 3. Managing language problems). Ethiopia is characterized by cultural and linguistic diversity. Though Amharic is both the country’s official language and the working language at TASH, many patients from rural areas often speak other languages (for example Oromo), and have problems understanding the staff. When communicating, both the physicians and the patients reported having to rely on other patients, staff, and family caregivers to manage interactions:

*Most of the patients come from the Oromia region, and most of us cannot communicate in Oromo*. *We try to use nurses, attendants or other patients to provide basic information. (Dr. female 5)*

Using family caregivers who can speak Amharic is a common option, though it is not always unproblematic. It is often both time consuming and stressful, as uncertainty concerning understanding and problems with expressing what is meant are reported:

*One of the problems I have here is language*. *I do not speak Amharic, I speak Oromo*. *Most of the doctors speak Amharic, so I have to always come with my family caregiver who can speak Amharic*. *There were instances where my family caregiver was not around and I had to deal with the doctor alone*. *That was too difficult, I must say*. *Even if your family caregiver or others around you can help you by interpreting, there is always a kind of misunderstanding and also it is so difficult to express your feelings when your family member is the one who is interpreting. (Pat. female 6)*

In relation to interpreting, in the video-recordings of hospital rounds patients were observed being “left outside” and family caregivers tended to dominate the interactions with the physicians. In the interviews, the physicians were aware of this problem, and often made attempts to involve the patients, as illustrated in Excerpt 1 below:

**Excerpt 1.**
**Doctor**: When did the disease begin?
**Family caregiver**: Hum… she…
**Doctor**: No, ask the patient.
**Family caregiver**: (Interprets from Oromo to Amharic)
**Patient:** Five months.

Here, the physician explicitly asked the family caregiver to interpret and ask the patient to answer the question herself.

Patient exclusions were observed in the interactions, when bad news was broken, as in Excerpt 2 below:

**Excerpt 2.**
**Doctor**: What was said there in the Gynecology Department about the uterus?
**Family caregiver:** She gave a sample from the uterus and we are waiting for an appointment to know the result.
**Doctor**: Um, were you not informed about going to the Radiotherapy Department for treatment?
**Patient**: No.
**Doctor**: (Turning to the family caregiver) Was the patient informed about cervical carcinoma?
**Family caregiver:** They informed only me.
**Doctor**: What about the patient?
**Family caregiver:** No, she doesn't know.

In this case, the patient could not interact with the doctor, because she hardly understood Amharic, and most of the communication was through the family caregiver. The excerpt illustrates that this patient was apparently not informed about her diagnosis, and the family caregiver was the only one who received the information. According to the physicians, patients, and family caregivers, bad news was often given to the family, rather than to the patients (Theme 4. Delivering bad news):

*It is inappropriate to tell patients that he/she has cancer*. *Instead of telling the patients tell their family caregivers. (Pat. female 33)*

A common reason for not telling the patient mentioned in the interviews is the belief that giving bad news can upset the patient, which is considered unnecessary in the case of a poor prognosis. The physicians, patients, and family caregivers agreed that it is the family that is often responsible for making decisions and taking responsibility for a patient’s care (Theme 5. Decision making). This is especially true for minors and women, and male family caregivers are often the decision makers:

*Usually, male family members are the ones who make decisions for the patients, especially if the patient is a child it’s often the father who makes the ultimate decision*. *In some cases, the doctors ask male family caregivers to leave to have a chance to communicate with the female patients. (Dr. female 2)*

Excerpt 3 below illustrates what was mentioned in the interview above. The physician explicitly asks the patient’s father to leave the room in order to talk to the female patient alone:

**Excerpt 3.**
**Doctor:** *(To the female patient)* Show me the medicines you have now. (*The doctor is shuffling the file of the patient)* There is something you haven’t told me, right?
**Family caregiver:** Who?
**Doctor:** The patient.
**Family caregiver:** For whom?
**Doctor:** *(Talking to the female patient)* There is something that you haven’t told me. *(Turning to the family caregiver)* Okay, you wait for me outside. Let me talk to her.
**Doctor:** (*Calling the patient by her name*) Are you married?
**Patient:** Yes.
**Doctor:** Who is your husband?
**Patient:** This is my father (*Pointing at her father who left*)

As the interaction proceeds, the physician finds out that the patient had previously had an abortion, which was probably difficult for her to discuss in front of her father. The doctor also realizes that the patient had not told him about the medicines she was taking:

**Excerpt 4.**
**Doctor:** This one was your first pregnancy?
**Patient:** Yes.
**Doctor:** Why did you take one round on the 8^th^ floor and then came here. You didn’t tell me. (*Looking at the patient*) Why didn’t you tell me that you took the first treatment there and the medication had worked?
**Patient:** I don’t know.
**Doctor:** Why did they send you back here?
**Patient:** I don’t know.

Excerpt 4 illustrates that the patient seems to experience problems understanding the treatment process. A possible reason could be a low awareness and knowledge of cancer, which, together with a low literacy level, complicates physician-patient communication (Theme 6. Illiteracy and cancer awareness):

*The major gap between physicians and our patients is literacy level*. *Most of the patients are illiterate… the other thing is cancer has become a more prominent disease in recent years*. *So most patients are not aware of it even if they are literate. (Dr. female 1)*

Illiteracy and lack of awareness of cancer, as well as living in rural areas far from hospitals often leads patients to seek professional help too late. The physicians mentioned that many patients come to TASH only after receiving treatments from traditional healers (Theme 7. Traditional and religious treatments). At this point, the cancer has often spread too far, and the physicians can only inform about poor prognosis and provide palliative care:

*Most of the patients who come from rural areas first try traditional medicine and if it does not work then they come to hospital*. *By the time they come the cancer has already spread. (Dr. male 1)*

In addition, as many Ethiopians are Orthodox Christians, one of their central beliefs is in the power of religion and holy water from the church to cure diseases:

*The Orthodox Christian patients often take holy water, and the problem is that they cannot combine medicines and holy water, so they discontinue medical treatment and take only holy water*. *If the holy water does not help them, they return to the hospital and usually they come when the cancer has progressed to the next stage. (Dr. male 3)*

In excerpt 5 below, the physician is talking to a patient who came to TASH two years after being diagnosed with cancer:

**Excerpt 5.**
**Doctor**: When did you get ill?
**Patient**: It was two years ago. I came here after two years.
(She leads his eyes to the other direction)
**Doctor**: Where did you get your diagnosis, is it here at Black Lion Hospital or at another place?
**Patient**: I was examined at Metehara (a town in central Ethiopia) and they told me it was cancer.
**Doctor**: What type of cancer?
**Patient**: Breast cancer, and they referred me to Tikur Anbessa, but some people told me that traditional medicine was good and I used it for two years. Then I came here.
**Doctor**: Did you observe any effects taking traditional medicine?
**Patient**: No change at all, and I got worse, and they referred me here (looking at the doctor).

The excerpt shows that the patient avoids looking at the physician while talking about coming to the hospital two years after being diagnosed with cancer and taking traditional medicines instead. It may indicate feelings of regret and being uncomfortable talking to the doctor about avoiding hospital care. Answering the question about the effects of traditional medicine, the patient is looking at the doctor, mentioning that she got worse, which possibly indicates her hope of getting help from the doctor.

According to the physicians, delays in receiving treatment were also related to stigmatization in relation to cancer (Theme 8. Stigmatization and fatalism). This also influenced their communication with patients. The physicians mentioned that the majority of female patients with breast and cervical cancer found it difficult to discuss their disease. For instance, in most parts of Ethiopia sex was something that people do not talk about openly in public or private, and women with cervical cancer were stigmatized. Talking about cancer also often lead to being isolated from family and society. Some people confused cancer with HIV, thinking that cancer is a communicable disease and so they avoid cancer patients. As a result, many patients asked physicians not to mention their cancer diagnosis:

*When patients request sick leave, they usually tell us not to mention the diagnosis because if their colleagues find out about their disease, they might exclude them or treat them differently*. *Patients with breast cancer have very limited sick leave annually but patients with HIV/Aids have unlimited sick leave. (Dr. male 8)*

Excerpt 6 below illustrates a patient trying to avoid spreading information about his condition and keeping the information even from close family members:

**Excerpt 6.**
**Doctor:** Did you tell your family that you have cancer?
**Patient:** I only told my wife umm…
**Doctor:** Didn’t you tell anybody else?
**Patient:** My wife and child. No one else knows. Neither my mother nor my father knows about my condition.
**Doctor.** How old is your child?
**Patient:** He is seven, going on eight.
**Doctor:** So you need to be strong, right?
**Patient:** Well, yes. What can you do if it’s God’s punishment? You just accept it.

The excerpt also illustrates the perception of cancer as God’s punishment and how the patient accepted it, which could potentially influence their willingness to undergo treatment.

## 4 Discussion

### 4.1 Principal findings

In this study, we present the communicative challenges in Ethiopian cancer care as perceived by physicians, patients, and family caregivers. Using the triangulation method in this research and combining data from semi-structured, in-depth interviews and video-recordings of authentic interactions have contributed to a better understanding of how the challenges are perceived and how communication problems are managed in interactions [35].

The results indicate that communication in Ethiopian oncology care is a challenge. Problems related to infrastructure, such as the availability of rooms for physician-patient-family caregiver communication, in combination with staff shortages, the volume of patients, and long distances between treatment facilities result in a challenging working environment [6, 36], which negatively influences communication.

Earlier studies in oncology provide evidence that time pressure negatively influences the quality and safety of patient care [37]. This study illustrates how physicians, due to time limitations, were unable to provide necessary information and give emotional support to patients and families while breaking bad news. Communication became additionally complicated when sensitive interactions were interrupted, due to a lack of private consultation rooms [38, 39]. In agreement with previous studies, our findings also showed that when the physicians and their patients spoke different languages (for example, Amharic and Oromo), interruptions were especially unfavorable, as they complicated understandings [40, 41]. All these factors created moral and ethical dilemmas with regard to the safety and efficacy of treatments, moral stress for the Ethiopian professionals, and frustration and uncertainty for the patients and their families.

The study adds to previous research describing a strong relationship between family and patient, which plays a paramount role in oncology care in Ethiopia in terms of informing patients about cancer [17], disclosure of diagnosis, experiences of stigma and fatalism [18], and in relation to decision-making and delivering bad news [24, 42]. In addition, the analysis of interviews and video-recordings both illustrate how family caregivers function as informal interpreters and advocates for patients in interactions with physicians not mentioned earlier in research about Ethiopian cancer care. Though this function is essential for managing consultations, this type of role for family members is known to have ethical implications [43, 44]. Our data indicates that family members consciously exclude patients from making decisions and prevent them from providing information to the physicians. The analysis of video-recorded interactions showed that patients with insufficient Amharic language skills were passive in consultations conducted in Amharic, and were dependent on their relatives’ help. Some physicians attempted to involve their patients in interactions by explicitly requesting family members to ask patients for information and to interpret their answers, instead of answering themselves. In addition, some patients were uncomfortable talking about their problems when family members were present (for example, in our study, a female patient talked about abortion only after the physician asked her father to leave the room). In such cases, it *is essential* for communication and treatment that the physician is able to accurately *detect* uneasiness and unwillingness to disclose sensitive information. This is especially important for female patients, as Ethiopia suffers from some of the lowest gender equality performance indicators in sub-Saharan Africa [45]. Many poorly educated or uneducated women do not participate in decision-making concerning their treatment, as male caregivers often make decisions on their behalf [46]. The findings, therefore, indicate that physicians face challenges considering their patients’ rights to privacy and confidentiality, due to the lack of professional interpreters and the considerable need for the support of family caregivers [47]. Further, having bilingual staff with clinical responsibility to interpret can be problematic, as they might not always maintain a neutral position when interpreting, have limited language knowledge, or uphold the patient’s right to confidentiality [44].

The findings of this study also indicate that family caregivers often avoid disclosing a cancer diagnosis to patients and insist that the physicians withhold information about diagnosis or poor treatment prognosis [42, 48]. In contrast, some patients also avoided revealing their diagnosis to their families. In the video-recorded interactions, a patient avoids telling his diagnosis to some members of his family. These attitudes are motivated by a strong wish not to upset family members, but also by a fear of social stigma and discrimination, as cancer is widely perceived as God’s punishment [6, 20, 49]. However, the practice impedes communication and contributes to a delay in obtaining healthcare and a diagnosis, adding to other factors revealed in our data and highlighted in other studies, such as illiteracy, lack of cancer awareness, limited knowledge about cancer, high travel costs to Addis Ababa, and preferences for traditional medicine [14, 17, 19, 50]. Due to unnecessary delays in diagnosis, many patients are diagnosed with an advanced disease that cannot be cured and can only be offered palliative treatment, which makes it additionally difficult for physicians to communicate the prognosis [14, 25].

While the general situation in Ethiopian cancer care is, of course, ethically challenging in its own right, the complexity around the role of family, combined with patients’ dependency on family members for communication, support, and access to care, creates particular ethical dilemmas for medical staff. This concerns what to tell patients and family members, and how to organize consultations to achieve patient-centered health communication, while maintaining a constructive alliance with the family and not jeopardizing the patient’s continued access to care.

### 4.2 Limitations

There is a risk that, in spite of assured anonymity, some patients and family caregivers were hesitant in expressing themselves freely, as they may have been anxious about jeopardizing their relationship with the physicians in case anything they said was divulged. As a result, we believe that some of the patients and family caregivers gave positive responses in order to maintain a good relationship with the physicians. Furthermore, even though the video camera was placed out of sight, being recorded could potentially have influenced the behavior of all the participants.

## 5 Conclusions and practice implications

The study highlights how communication in Ethiopian cancer care can be negatively influenced by socio-economic and cultural factors. Assuming that the surrounding structural contexts of Ethiopian cancer care will not change rapidly, there is a need for organizational development to better meet the communicative challenges that exist. In addition, strategies to help respond to this situation may include that staff, such as nurses or lay patient counsellors, are given more health communicative responsibilities, and that logistical ways for staff to speak to patients independently of the family are developed. At another level, future professionals need better health communication training (for example, through including such skills in training oncology specialist authorization for doctors and nurses). The delicate ethical dilemmas related to balancing patient and family communication, and the related decision-making authority, may require special guidelines that accommodate the unique challenges in Ethiopian cancer care. A greater focus on the ethical dilemmas of managing communication when a family member asks to withhold vital information from a patient is needed, as refusing to comply may impede the patient’s ongoing access to healthcare. The integration of communication training for medical students in Ethiopia, with a focus on an ethical guideline for family-centered patient consultation suitable for these circumstances, would be an essential step.

## Supporting information

S1 Appendix (DOCX)
