# Supplementary material for: Communicative challenges among physicians, patients, and family caregivers in cancer care: An exploratory qualitative study in Ethiopia
Source: PLoS One. 2020 Mar 13;15(3):e0230309. doi: 10.1371/journal.pone.0230309 (PMC7069641; doi:10.1371/journal.pone.0230309)
Supplement: S1 Appendix — (DOCX) [file pone.0230309.s001.docx]

**Interview guide: physician**

*Thank you for participating in our interview. We would like to ask you some questions about your communication with patients and their family caregivers.*

1. How many years have you worked as a doctor with cancer patients?
2. What kinds of cancer do your patients have?
3. Do they usually come alone, or with family members?
4. How do you experience communicating with cancer patients? What do you think works well and what is problematic?
5. What do you think hinders effective communication between doctors and patients and family/caregivers, and how do you overcome these problems?
6. How do go about breaking bad news to patients? Do you tell patients, family or both?
7. Who usually makes a decision about treatment?
8. Do you have anything to add?

**Interview guide: family/ caregiver**

*Thank you for participating in our interview. We understand that you have a relative/patient with cancer. We would like to ask you some questions about your involvement in your relative’s/patient’s cancer treatment and communication with doctors.*

1. What kind of cancer does your relative/patient have?
2. Do you usually accompany your relative/patient to the doctor?
3. How do you experience communicating with doctors?
4. What do you think works well, and what is more difficult or problematic?
5. How did you know about your relative’s/patient’s diagnosis? Who told you about this?
6. Who makes decisions about treatment?
7. Do you have anything to add?

**Interview guide: patient**

*Thank you for participating in our interview. We would like to ask you some questions about the way doctors communicate with you concerning your condition and treatment.*

1. Did you come alone? With family members? Friends?
2. How do you experience communicating with doctors?
3. What do you think works well, and what is more difficult or problematic?
4. How did you know about your diagnosis? Who told you about this?
5. Who makes decisions about treatment?
6. Do you have anything to add?
